# A prospective study of ‘circumferential purse-string approximation’ vs. primary linear skin closure in stoma reversal

**DOI:** 10.11604/pamj.2022.42.287.29213

**Published:** 2022-08-17

**Authors:** Manavdeep Singh Bains, Amandeep Singh Nar, Harmandeep Singh Jabbal, Atul Mishra, Akashi Mishra, Priyanka Sharma

**Affiliations:** 1 Bains General and Surgical Hospital, Nawanshahar, Punjab, India,; 2 Department of General Surgery, Dayanand Medical College and Hospital, Ludhiana, Punjab, India,; 3 Department of General Surgery, All India Institute of Medical Sciences, Bathinda, Punjab, India,; 4 Himalayan Institute of Medical Sciences, Jolly Grant, Dehradun, Uttrakhand, India,; 5 Department of Pathology, Adesh Institute of Medical Sciences and Research, Bathinda, Punjab, India

**Keywords:** Stoma, closure, reversal, technique, circumferential purse-string approximation, primary linear closure, surgical site infection

## Abstract

**Introduction:**

surgical site infection (SSI) is one of the most common complications that can occur after stoma closure. To date, there is no consensus on the ideal closure technique of the stoma wound to minimize postoperative SSI and multiple techniques have been proposed. We performed this study to assess the clinical outcome of wound healing after ‘Primary Linear Closure’ (PC) and ‘Circumferential Purse-String Approximation’ (CPA) techniques.

**Methods:**

this prospective observational study included all patients admitted to our tertiary care center, fulfilling the inclusion criteria for elective stoma closure from 1^st^ March 2018 to 1 March 2020 and prospective study was conducted on wound healing after stoma closure to compare difference in SSI rate between the PC group and the CPA group and to discuss the differences in patient satisfaction with wound healing. The following study was carried out with 36 patients in purse-string group and 66 patients in linear closure group.

**Results:**

surgical site infection (SSI) was observed only in primary linear closure group in 24% patients as opposed to 0% in purse string closure group. (p=0.039). Although the mean healing time of wound in linear closure group was significantly less than the purse string group (10.76 ± 5.68 days and 14.17 ± 2.04 days respectively), the overall total satisfaction score was higher in the purse string group. The purse string closure group showed significantly higher satisfaction score for expectations regarding appearance of scar/cosmesis, level of postoperative pain and difficulty of wound care (p>0.05).

**Conclusion:**

in our study, ‘Circumferential Purse-String Approximation’ is a superior technique than Primary Linear Closure for Stoma reversal in terms of rate of SSI and better overall patient satisfaction. CPA is a good alternative option, but further prospective randomized trials involving more patients are necessary before a definitive conclusion can be drawn.

## Introduction

A temporary stoma is frequently used in the treatment of intestinal perforation, obstruction, colorectal cancer, inflammatory bowel disease and diverticulitis. In particular, it is used to reduce the anastomotic leakage and the reoperation rate. Various complications have been reported in literature after stoma closure. Wound infection, small bowel obstruction, anastomotic leakage and incisional hernia are some of the well-known complications [1]. Surgical site infection (SSI) is one of the most common complications that can occur after stoma closure. The incidence of wound infection ranges from 0% to as high as 41% [2,3]. The potential for infection depends on a number of patient variables such as the state of hydration, nutrition, existing medical conditions as well as extrinsic factors related to pre, intra and post operative care, the skin closure technique, sterility maintained intra-operatively and post operative wound care management. The common causes of wound infection are wound contamination due to bacteria on the skin in the vicinity of the ileostomy in contact with bowel contents for a long time and the leakage of the ileostomy contents. It is associated with increased costs for healthcare services, hospital stays, medications, and nursery care, as well as with increased morbidity and poor quality of life [1,2,4]. A number of skin closure techniques have been tried to minimize postoperative SSI but till now there is no consensus on the ideal closure technique for a stoma wound. Multiple techniques have been proposed like tight primary closure, loose primary closure, delayed primary closure, or no closure and healing by secondary intention. A hybrid technique was proposed by Banerjee [4], in which he performed a purse string skin closure of wound and observed less wound infection and smaller scars thus making it cosmetically superior. This technique combines the concept of leaving the wound open to provide drainage and minimize SSI while still providing some degree of wound opposition to minimize healing time. Until now, very few studies analyzed the effectiveness of purse-string skin closure in comparison to other methods of wound closure following stoma reversal. Studies analyzing the effectiveness of purse-string skin closure following stoma closure are limited but have shown promising results in minimizing SSIs. Hence, this study was conducted with the objective to assess the efficacy of purse-string skin closure in an ileostomy reversal as compared to conventional linear skin closure in decreasing the rates of wound infection.

## Methods

**Study Setting**: this prospective observational study was conducted at Dayanand Medical College and Hospital (DMC & H), Ludhiana, India and included all patients admitted for elective stoma closure from 1^st^ march 2018 to 1^st^ march 2020.

**Study design**: prospective observational cohort study.

**Study participants**: one hunderd and two patients enrolled into the study.

**Inclusion criteria**: all patients with status stoma in situ admitted to DMC & H, Ludhiana for elective stoma closure.

**Exclusion criteria**: 1) extreme of age >70 and < 15 years. 2) Those with immune-compromised state. 3) Patients not willing to give consent for participation in this study.

### Recruitment of samples

**Informed consent**: was taken from all patients participating in the study.

**The present study was carried out after simple random allocation of patients to two groups**: 1) patients undergoing Primary Linear Closure (PC); 2) patients undergoing Circumferential Purse-String Approximation (CPA).

In the PC group, an elliptical skin incision was made to mobilize the stoma from abdominal wall followed by anastomosis of protruding bowel. The sheath at the stoma site was closed with an absorbable multifilament-interrupted suture. A non-absorbable monofilament material was used to close the peri-stomal incision. No wound drainage was applied for any of the patient who underwent a PC. In the CPA group, a circular incision was made at the Entero-cutaneous junction. The bowel anastomosis and the sheath closure were same as that in PC group. A purse-string sub-cuticular suture (absorbable 2/0 multifilament/monofilament) was used to approximate the skin circumferentially leaving only a small defect of approximately 1-1.5 cm diameter in the center.

A sterile dressing was applied using a loose packing of povidine-iodine gauze. Daily dressing was done in both groups. Any wound, if infected, was opened, collection drained and cavity packed with gauze daily. Wound swab was taken and sent for culture and sensitivity test and antibiotics started according to culture/sensitivity report. The time to complete healing was defined as time at which a dressing was no longer needed after stitch removal and the patient no longer needed medication for wound discomfort. In addition, a questionnaire (The Korean version of patient wound healing satisfaction scale) [5] was used to quantify and compare the differences in patient reported wound healing satisfaction and cosmesis after complete wound healing. This scale is five point Likert-Type scale. A score of 1 to 5 was assigned for each factor, with higher scores indicating increased satisfaction. Factors assessed include patient satisfaction regarding appearance of the scar, level of postoperative pain, time of wound healing, difficulty of wound care and limitation of activity.

**Statistical analysis**: data was described in terms of range; mean ± standard deviation (± SD), median, frequencies (number of cases) and relative frequencies (percentages) as appropriate. Comparison of quantitative variables between the study groups was done using Student t-test. For comparing categorical data, Chi square (χ^2^) test was performed and exact test was used when the expected frequency is less than 5. Statistical analysis was done using GraphPad Prism version 5.0 (GraphPad Software, Inc., LaJolla, CA, USA) and SPSS 22.0 software (SPSS, Inc., Chicago, IL, USA). P<0.05 was considered statistically significant.

**Ethical clearance**: was taken from Research and Ethical Clearance Committee, Dayanand Medical College and Hospital, Ludhiana.

## Results

A hospital-based prospective observational study was conducted on wound healing after stoma closure to compare difference in SSI rate between the PC (primary linear closure) group and the CPA (circumferential purse-string approximation) group and to discuss the differences in patient satisfaction with wound healing. The following study was carried out with 36 patients in purse-string group and 66 patients in linear closure group. The 102 patients studied belonged to the northwestern region of India. Both groups were followed up for minimum 3 months´ duration.

**Comparison of mean duration of operative time**: the mean duration of operative time in linear closure group and purse-string closure group was 1.25 ± 0.13 and 1.20 ± 0.07 hours respectively. There was no significant difference between the groups as per Student t- test (p>0.05) (Table 1).

**Table 1:** operative time, patients satisfaction score according to cosmesis, pain, healing time, limitation of activity, wound care and incidence of SSI

Variables	PC		CPA		t-test	p-value
	Mean	SD	Mean	SD		
**Operative time (hours)**	1.25	0.13	1.20	0.07	1.446	0.153
**Cosmesis**	3.21	0.73	4.78	0.42	-11.765	0.000
**Pain**	3.27	0.87	4.61	0.49	-8.508	0.000
**Healing time**	3.21	0.85	3.39	0.49	-1.144	0.255
**Limitation of activity**	3.33	0.88	3.56	0.50	-1.390	0.168
**Wound care**	3.03	0.80	4.61	0.49	-10.737	0.000
	**PC** (16/66)		**CPA** (0/36)		**Chi-square value**	**p-value**
Surgical site infection (**SSI)**	24%		0%		5.175	0.039

**Distribution of patients according to complications (surgical site infection)**: the complication rate in linear closure group and purse-string closure group was 24% (16 out of 66) and 0% (0 out of 36) respectively. There was a significant difference between the groups as per Student t-test (p<0.05) (Table 1).

**Figure 1:**
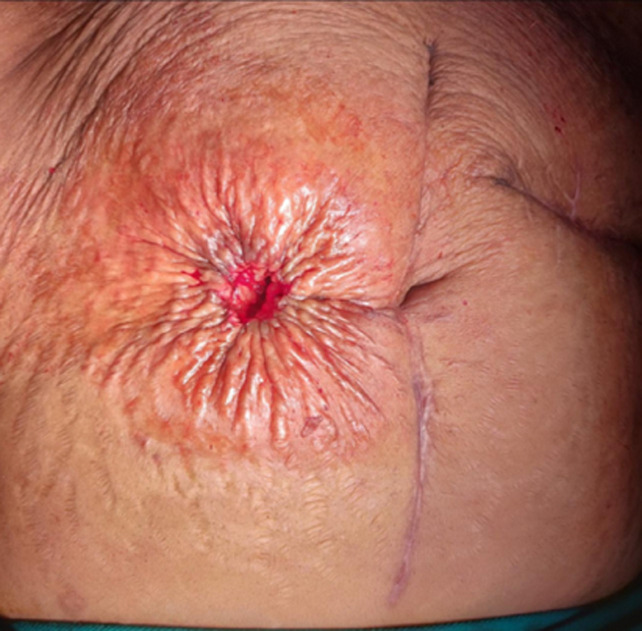
‘circumferential purse-string approximation’ with 1.5 cm defect

**Distribution of patients satisfaction score according to cosmesis, pain, healing time, limitation of activity and wound care**: the overall total satisfaction score was higher in the purse string group. The purse string closure group showed significantly higher satisfaction score for expectations regarding appearance of scar/cosmesis, level of postoperative pain and difficulty of wound care. There was significant difference between the groups as per Student t-test (p<0.05). There were no significant difference in patients satisfaction score for time of wound healing and limitation of activities (p>0.05) (Table 1, Figure 1).

**Distribution of patients according to hospital stay and healing time**: the mean duration of Hospital Stay in linear closure group and purse-string closure group was 7.82 ± 1.69 and 7.78 ± 1.46 days respectively. There was no significant difference between the groups as per Student t-test (p>0.05). The mean healing time of wound in linear closure group and purse string group was 10.76 ± 5.68 days and 14.17 ± 2.04 days respectively. There was significant difference between the groups as per Student t-test (p<0.05) (Table 2).

**Table 2:** distribution of patients according to hospital stay and healing time

Variables	PC		CPA		t - test	p-value
	Mean	SD	Mean	SD		
**length of stay (days)**	7.82	1.65	7.78	1.48	0.322	0.749
**Healing time (days)**	10.76	5.68	14.17	2.04	-2.432	0.018
**Without SSI**	7.76	1.52	-	-	-19.741	0.000
**With SSI**	20.13	2.93	-	-		

## Discussion

The mean duration of operative time in linear closure group and purse-string closure group was 1.25 ± 0.13 and 1.20 ± 0.07 hours respectively. There was no significant difference between the groups as per Student t-test (p>0.05) (Table 1). Marquez *et al*. [6] conducted a study duration of surgery was higher in the CPA (1.41 hours) group than it was in the PC (1.17 hours) group, but the difference was not statistically significant (P = 0.19). Camacho-Mauries *et al*. [7] study reported duration of surgery was higher in the CPA (2.11 hours) group than it was in the PC (2.03 ± 9 hours) group, but the difference was not statistically significant (P = 0.733). Mina Alvandipour *et al*. [8] conducted a randomized clinical trial in which duration of surgery was higher in the purse-string closure (PSC) (1.11 ± 9 hours) group than it was in the PC (1.06 ± 9 hours) group, but the difference was not statistically significant (P = 0.416). O’Leary [9] conducted a randomized, controlled trial and concluded that the operative time was shorter in the PC group, but this was not statistically significant (63.1 vs. 71 min, p = 0.28).

In this prospective nonrandomized trial, the SSI rate was 24% (16 out of 66 patients) after primary linear closure and 0% (0 out of 36) SSI occurred in the CPA group (Table 1). There was a significant difference between the groups as per Student t-test (p<0.05). This is comparable to the studies of Alvandipour *et al*. [8], Sutton *et al*. [10], Gachabayov *et al*. [11] and Yoon *et al*. [12]. Alvandipour *et al*. [8] conducted a randomized clinical trial. Infection occurred in 1 of 34 CPA patients (2.9%) and in 7 of 32 PC patients (21.8%). Sutton *et al*. [10] reported that in their study, 0% SSI occurred in 51 patients (38 men, 13 women; average age 64 years, age range 52-81 years) who underwent a purse-string approximation. Gachabayov *et al*. [11] conducted a systematic review and meta-analysis of twenty studies (6 experimental and 14 observational) including 1812 patients (826 CPA and 986 PC). SSI rates were significantly lower statistically and clinically in patients with CPA in the meta-analysis of all studies. SSI rate was 3.1% (26/826) in CPA vs. 20.2% (199/986) in PC. Yoon *et al*. [12] conducted a prospective nonrandomized trial, 48 patients of two groups CPA group (n = 34) and the PC group (n = 14) who underwent a stoma closure. CPA was associated with a significantly lower incidence of wound infection (3/14 [21.4%] in PC vs. 0/34 [0%] in CPA, which is very similar to our study.

In our study, there was a difference in the time to complete healing between the PC and the CPA groups. The mean healing time of wound in linear closure group and purse string group was 10.76 ± 5.68 days and 14.17 ± 2.04 days respectively. There was a significant difference between the groups as per Student t-test (p<0.05) (Table 2). The mean healing time of wound in linear closure without and with SSI was 7.76 ± 1.52 days and 20.13 ± 2.93 days respectively (Table 2). There was significant difference between the groups as per Student t-test (p<0.05). Wada *et al*. [13] conducted a retrospective study on total of 55 consecutive patients undergoing purse-string skin closure (26 patients) and conventional linear skin closure with a drainage tube (29 patients) following stoma closure. He found time to complete healing in linear closure group (13 days, range 8-29 days) was shorter than the purse string group (14 days, range 9-50 days). Camacho-Mauries *et al*. [7] reported healing time was also different; it was 40-45 days in the linear closure group with SSI and 25-28 days in the purse-string group (p< 0.0001). He also found that subgroup of patients who did not have an infection in linear closure group had a shorter healing time of 2 weeks (18-22 days), which is approximately half the healing time in the purse-string group in his study.

**Figure 2:**
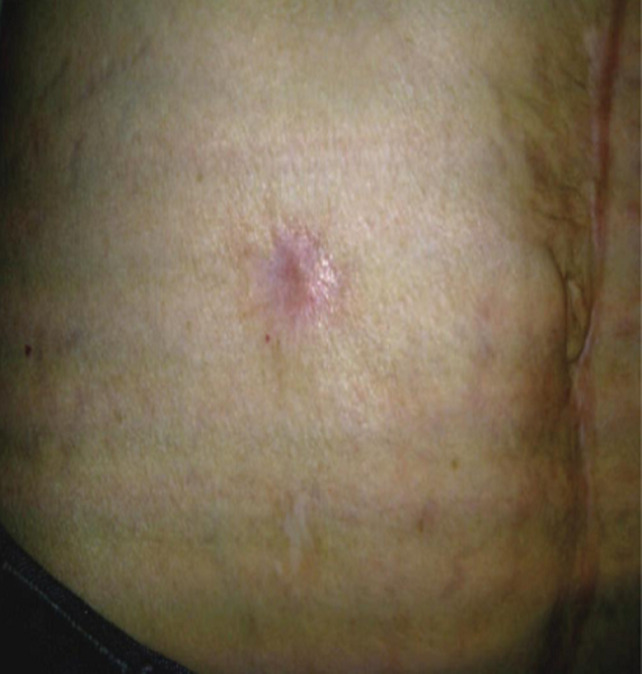
minimal scar after complete healing at 1 year

The mean duration of hospital stay in linear closure group and purse-string closure group was 7.82 ± 1.65 and 7.78 ± 1.48 days respectively (Table 2). There was no significant difference between the groups as per Student t-test (p>0.05). Marquez *et al*. [6] study showed mean duration of hospital stay after surgery was higher in the CPA [6 days] group than it was in the PC group [5 days], but the difference was not statistically significant (P = 0.51). Camacho-Mauries *et al*. [7] study had mean hospital stays of 8.4 and 7.2 days in the CPA and the PC group, respectively, but this difference was not statistically significant (P = 0.429). The overall total satisfaction score was higher in the purse string group (Table 1). The purse string closure group showed significantly higher satisfaction score for expectations regarding appearance of scar/cosmesis, level of postoperative pain and difficulty of wound care. There was significant difference between the groups as per Student t-test (p<0.05). There were no significant difference in patients satisfaction score for time of wound healing and limitation of activities (p>0.05). Milanchi *et al*. [5] study reported a trend toward better cosmetic results for CPA than for PC. He also reported a significantly higher mean patient satisfaction score in the circumferential subcuticular wound approximation group than in the PC group. In Klink *et al*. [14] study, patients who underwent a CPA found that while the initial circular scar might be unappealing, final scar formation occurred along natural skin tension lines, producing a cosmetically pleasant scar (Figure 1, Figure 2). With the purse-string procedure, level of postoperative pain is less as until granulation tissues grow and the skin is epithelialized, small skin defect areas become natural drainage pathways, which continuously drains seroma fluid which prevents any oedema, tenderness and wound infection. Also minimal handling and no need of any poking of wound in postop dressings minimize the pain. Patients' responses and significant differences in our study indicated that the CPA was associated with less postoperative pain and discomfort.

## Conclusion

In our study, ‘Circumferential Purse-String Approximation’ is a superior technique than primary linear closure for Stoma reversal with better overall outcomes. Incidence of SSI after CPA was lower than it was when a PC was used. Also, patient satisfaction for expectations regarding appearance of scar/cosmesis, level of postoperative pain and ease of wound care was greater in the CPA group than in the PC group. However, the CPA technique takes a longer time to heal than the PC technique. We conclude that CPA is a good alternative option for stoma reversal, but due to some limitations of non-randomized and small sample size, further prospective randomized trials involving more patients are necessary before a definitive conclusion can be drawn.

### What is known about this topic

- Stoma reversal with Primary Linear Skin Closure has a significant rate of SSI;
- Patient concern for cosmesis, pain and overall satisfaction post reversal of stoma.

### What this study adds

- Circumferential Purse-String Approximation in a viable alternative for Stoma reversal;
- Cosmesis, pain scores and overall patient satisfaction are better with CPA than PC.
